# Detection of anti-*Toxoplasma gondii* antibodies in chronic myeloid leukemia and acute myeloid leukemia patients

**DOI:** 10.14202/vetworld.2017.1063-1065

**Published:** 2017-09-13

**Authors:** Mohammad Javad Gharavi, Mona Roozbehani, Zienat Mandeh

**Affiliations:** 1 Department of Parasitology, Faculty of Allied Medicine, Iran University of Medical Sciences, Tehran, Iran; 2 Department of Parasitology, School of Medicine, Iran University of Medical Sciences, Tehran, Iran

**Keywords:** acute myeloid leukemia, chronic myeloid leukemia, *Toxoplasma gondii*

## Abstract

**IN:**

**Background and Aim:** Infection of *Toxoplasma gondii* is a worldwide distribution. Toxoplasmosis in patients who are immunocompromised by virtue of underlying leukemia disease has received relatively little attention. This study was aimed to evaluate IgG and IgM antibodies of *T. gondii* and to minimize the role of *T. gondii* and opportunistic infection complication at the early stage of infection in leukemia patients.

**Materials and Methods:**

The purpose of this assay was to measure anti-*T. gondii* IgG and IgM antibodies by enzyme-linked immunosorbent assay (ELISA) technique in leukemia patients.

**Results:**

IgG antibodies against *T. gondii* were detected by ELISA in 96 (56.4%) leukemia patients and 72 (42.4%) control group. IgM antibodies were found in 10 patients (5.9%) with leukemia and 3 (1.8%) in the corresponding.

**Conclusion:**

Our finding indicated that leukemia patients under immunosuppressive condition should not be neglected. Toxoplasmosis in leukemia patients as a main risk factor is considered, meanwhile in some patients, due to possibility of the presence of secondary infection that leads to severe toxoplasmosis.

## Introduction

*Toxoplasma gondii* is a zoonotic coccidian obligate, intracellular protozoan parasite, which humans and other warm-blooded animals are its hosts [1]. The infection has a worldwide distribution, and the incidence of the disease varies around the world [2]. Humans get *T. gondii* through ingestion of undercooked meat, contact with feline feces, and sometimes through drinking contaminated water, food, and vegetables or through transplantation of infected organ [3]. Human toxoplasmosis is often subclinical or with slight symptom [4]. Moreover, the parasite’s bradyzoites can persist inside human cells for long time periods, but recent infection can be reactivated, such as in the case of AIDS which *T. gondii* reactivation causes severe encephalitis [5]. In this regard, the prevalence of *T. gondii* can induced encephalitis to reach up to 40% in patients with AIDS in which 10-30% die from this parasitic disease [6].

This protozoan has occasionally observed in patients with neoplasias or transplantation recipients who are under immunosuppressive therapy. In these patients, the disease resulted in the reactivation of chronic infection. The acute infection, in patients with disseminated disease, often involves the central nervous system, with diffuse encephalopathy and meningoencephalitis including cerebral mass lesions [7,8]. Toxoplasmosis in patients who are immunocompromised by virtue of underlying leukemia disease has received relatively little attention. Leukemia is a disease resulting from the neoplastic proliferation of hemopoietic including lymphoid cells. It results from mutation, or epigenetic factors lead into clonal expansion. The clinical advent is flaw cells that are usually, directly, or indirectly, to the proliferation [9].

Drug induces immunosuppressive leukemia patients, to assess the risk of secondary severe toxoplasmosis. Therefore, This study was aimed to evaluate IgG and IgM antibodies of *T. gondii* and to minimize the role of *T. gondii* and opportunistic infection complication at the early stage of infection in leukemia patients.

## Materials and Methods

### Ethical approval

This project underwent ethical review and was approved by the Ethics Committee of Iran University of Medical Sciences.

### Subjects

A cross-sectional serosurvey of *T. gondii* antibodies in 170 (65 men and 105 women) patients with leukemia disorders from the Oncology and Haematology Department, Shariati Hospital, Tehran, Iran, and Fardis Central Laboratory, in Alborz Province of Iran, between October 2014 and March 2015, in parallel 170 healthy volunteers with identical age and sex match individual, were selected as corresponding control. Our samples consisted of both chronic myeloid leukemia (CML) and acute myeloid leukemia (AML). Peripheral blood was taken from all leukemia patients and control group under aseptic conditions, and the sera were separated and stored at −20°C without any discrepancy.

### Serological technique

Enzyme-linked immunosorbent assay (ELISA) was used for the assessment of anti-*T. gondii* IgG and IgM antibodies. The ELISA kit was provided by Euroimmun, a commercial manufacturer in Germany. The test performed base on the manufacturer’s guideline. The absorbance of serum samples was divided into the absorbance of the low calibrator. The ratio above 1/1 was considered as positive for both IgM and IgG antibodies, respectively.

### Statistical analysis

The statistical evaluation was based on Chi-squared test by SPSS version 16 for Windows pocket program. A p<0.05 was considered as statistically significant.

## Results

In this study, the age of leukemia patients was 10-60 years old. Table-1 summarized the distribution of CML and AML myelogenous leukemia patients. 38% of patients were male and 62% of patients were female. *T. gondii* IgG antibodies were assessed by ELISA in 96 (56.4%) leukemia patients and 72 (42.4%) control group. IgM antibodies were detected in 10 leukemia patients (5.9%) and 3 (1.8%) in control group. The seroprevalence distributions of the two groups were shown in Table-2. IgG antibodies of *T. gondii* were found in 40 (41.6%) male and 56 (58.4%) female leukemia patients. IgM antibodies of *T. gondii* were found in 3 (30%) male and 7 (70%) female leukemia patients. The difference in the IgG seropositivity rate between the patient and the control group is statistically significant (p<0.05). The seropositivity rate of anti-*T. gondii* IgM was higher in the patients group and statistically significant (p<0.05) (Table-2).

**Table-1:** Patients age distribution.

Age groups (years)	n		n (%)	
	
	CML	AML	Frequency patients	Frequency healthy volunteers
11-20	4	21	25 (14.7)	22 (12.9)
21-30	7	32	39 (22.9)	35 (20.6)
31-40	8	23	31 (18.2)	30 (17.6)
41-50	16	12	28 (16.4)	32 (18.8)
51-60	20	11	31 (18.2)	33 (19.4)
>60	12	4	16 (9.4)	18 (10.6)
Total	67	103	170 (100)	170 (100)

CML=Chronic myeloid leukemia, AML=Acute myeloid leukemia

**Table-2:** The result of serosurvey for *T. gondii* antibodies of leukemic patients and the control group.

Antibody	Leukemic patients n (%)			Control group n (%)		
	
	n=170	Male	Female	n=170	Male	Female
Anti-*T. gondii* IgG	96 (56.4)	40 (41.6)	56 (58.4)	72 (42.4)	30 (41.6)	42 (58.3)
Anti-*T. gondii* IgM	10 (5.9)	3 (30)	7 (70)	3 (1.8)	1 (33.3)	2 (66.6)

*T. gondii=Toxoplasma gondii*, IgG=Immunoglobulin G, IgM=Immunoglobulin M

## Discussion

One of the most critical problems in leukemia is infectious diseases which may lead the patient succumbs to sudden death. The active infection may alter the normal immune response of the host. Granulocytes and macrophages play a main role immune surveillance in innate immune system [10]. In this study, immunocompromised patients were found more frequently infected with *T. gondii* revealed by seropositivity both IgG and IgM anti-*T. gondii* in compare to corresponding control (p<0.05).

In fact, leukemia patients demonstrated that IgM and IgG anti-*T. gondii* antibodies were 10 (5.9%) and 96 (56.4%), respectively. In line with, control group, result showed IgM and IgG anti-*T.gondii* antibodies were 3(1.8%) and 72 (42.4%). It is in trigging to note the level of IgG and IgM antibodies were consistently moderate higher in leukemia patients than control groups (p<0.05). This is indicative of possible reactivation of *T. gondii* infection in leukemia patients.

This is in compromise with the studies reported by Yazar *et al*. [11] in which patients neoplasia demonstrate superior value of *T. gondii* IgG and IgM antibodies seropositive (52.9%) considered the control group (20%) [11].

Furthermore, other research showed *T. gondii* IgG antibodies in 114 (45.2%) cancer patients were positive, and *T. gondii* IgM+ antibodies were 26 (10.3%) as well as 17 (6.7%) of specimens had both IgG and IgM antibodies. In control group, 92 (36.5%) cases and 15 (6%) cases revealed seropositive for IgG and IgM antibodies, respectively [12].

Furthermore, Gharavi *et al*. [13] assessed IgM and IgG anti-*T. gondii* in renal transplant recipients, before and after transplantation. ELFA method detected 65 (63.7%) pre-transplantation specimens as IgG+ and did not detect any IgM+. Nevertheless, IgM was detected in 3 (2.9%) post-transplantation samples.

Moreover, additional studies indicated that the difference frequency of positive IgG antibodies between the patients with cancer and the control group was significant, whereas the positivity rates of IgM did not show any significant difference [6].

These findings may suggest that leukemic patients under immunosuppressive condition had been infected with *T. gondii* before initiation of leukemia development. Therefore, the immunosuppressive therapy may provide an opportunity for the inactive infection to undergo to full-blown toxoplasma disease. According to our finding, leukemic patients as a group of patients being treated with immunosuppressed drugs should not be neglected. However, further studies such as antigen detection and gene amplification (polymerase chain reaction) may facilitate to elucidate the activity of *T. gondii* in immunocompromised patients.

## Authors’ Contributions

MR and MJGH have designed the concept and supervised the plan of work and also have prepared the manuscript. ZM has contributed in sample collection, and technical methods. MR and MJGH have analyzed and interpreted the data. All authors read and approved the final manuscript.
